# Gap-directed chemical lift-off lithographic nanoarchitectonics for arbitrary sub-micrometer patterning

**DOI:** 10.3762/bjnano.14.4

**Published:** 2023-01-04

**Authors:** Chang-Ming Wang, Hong-Sheng Chan, Chia-Li Liao, Che-Wei Chang, Wei-Ssu Liao

**Affiliations:** 1 Department of Chemistry, National Taiwan University, Taipei 10617, Taiwan

**Keywords:** chemical lift-off lithography, gap, self-assembled monolayer, sub-micrometer, surface patterning

## Abstract

We introduce a unique soft lithographic operation that exploits stamp roof collapse-induced gaps to selectively remove an alkanethiol self-assembled monolayer (SAM) on Au to generate surface patterns that are orders of magnitude smaller than structures on the original elastomer stamp. The smallest achieved feature dimension is 5 nm using a micrometer-scale structured stamp in a chemical lift-off lithography (CLL) process. Molecular patterns retained in the gaps between stamp features and their circumscribed or inscribed circles follow mathematical predictions, and their sizes can be tuned by altering the stamp structure dimensions, including height, pitch, and shape. These generated surface molecular patterns can function as biorecognition arrays or be transferred to the underneath Au layer for metallic structure creation. By combining CLL process with this gap phenomenon, soft material properties that are previously thought as demerits can be used to achieve sub-10 nm features in a straightforward sketch.

## Introduction

The development of lithographic techniques is crucial to the advancement of the electronics and semiconductor industry, the backbones of modern technology. Advances in photolithography have pushed the limit of commercially available techniques down to the 10 nm scale in the form of extreme ultraviolet lithography [1–2], though equipment cost and energy consumption substantially increase with smaller desired feature dimension. On the contrary, direct-write methods like electron beam lithography can be used to produce even smaller features with arbitrary shapes, but the serial nature of these practices forbids their suitableness for high volume productions [3–4]. Soft lithographic techniques are hybrid approaches which have been extensively studied as alternatives to achieve precise patterning through reusable economic hardware and straightforward processes [5]. For instance, microcontact printing, which transfers ink molecules (e.g., alkanethiols) onto a surface (e.g., Au) using a soft material stamp (e.g., polydimethylsiloxane), is a strong contender in this field as the technique is easy to perform, robust to operate, and inexpensive to conduct [6–7]. With diverse compatible ink choices ranging from simple organic molecules [6] to silicones [8], proteins [9–10], DNA [11], and living cells [12], microcontact-printing-correlated techniques have shown great potential in biochemical research and device fabrication [13–14]. Nevertheless, pattern resolution and reproducibility in contact printing approaches are affected by several factors, most notably the ink molecule lateral diffusion, gas phase transportation, and rubber stamp deformation [15–16]. These are unavoidable issues in soft lithography operations and could severely limit the obtainable feature resolution if neglected.

Chemical lift-off lithography (CLL) is a rapidly emerging subtractive lithographic technique that aims to overcome the lateral diffusion and gas phase transfer obstacles present in conventional soft lithography [17–19]. Instead of transferring ink molecules from a stamp reservoir, this technique selectively removes alkanethiol molecules from a self-assembled monolayer (SAM) covered flat Au surface. An oxygen-plasma-activated polydimethylsiloxane (PDMS) stamp bearing patterns originating from a silicon master mold is placed in contact with the SAM, which initiates a covalent bonding between stamp surface siloxyl groups and alkanethiol hydroxy tails. Removal of the stamp from the SAM-covered surface lifts off alkanethiol molecules at the contact region, and thus circumventing the lateral diffusion issue to allow high-resolution patterning over a large area. In addition to standard lithographic operations using this approach, the CLL process can also be applied to create functional molecular patterns by backfilling post lift-off regions with various molecules [20–22]. Interestingly, the CLL process provides a unique artificial defect-rich surface, which supports the creation of homogeneously distributed molecular environments for functional substrates, e.g., surface wettability control [23] and enhanced biorecognition phenomenon [24–26]. The versatility of CLL can also be expanded by modulating stamp properties for micrometer-scale features [27]. utilizing different assembled and backfilled species [28–29]. and further substrate processing, e.g., pattern transfer to the underlying material layer [30–36]. In practice, CLL allows simple and facile fabrication of nanoscale surface patterns, whereas conventional photolithography methods are limited by diffraction during the illumination step and under/overetching during the development process [17].

In soft lithography, the deformation and collapsing of rubber stamp structures occur due to the adhesion between the stamp and the substrate, and are viewed as nuisances that hinder technique resolution and reproducibility [37–42]. Careful treatments on stamp feature design and aspect ratio tuning are therefore necessary to achieve desirable patterning results. Although this problematic issue may cause lithographic limitation, the structural gaps generated at the stamp–substrate interface during the contacting stage can provide another opportunity to create minute geometries. For example, nanochannels with height on the order of 10 nm and millimeters in length can be created when a nanowire is placed in the gap between the supporting substrate and a capping layer [43–45]. On the other hand, capillary force can induce the formation of nanochannel gaps when a structural top layer is brought into contact with the bottom surface [43]. Through these techniques, structures that are at the nanometer level can be obtained via the use of microscale features generated from conventional lithography. Nevertheless, the integration of interface structure gap formation and soft lithography, unfortunately, is still challenging since the molecular lateral diffusion problem remains to restrict pattern resolution especially when a tiny gap is created.

**Scheme 1:**
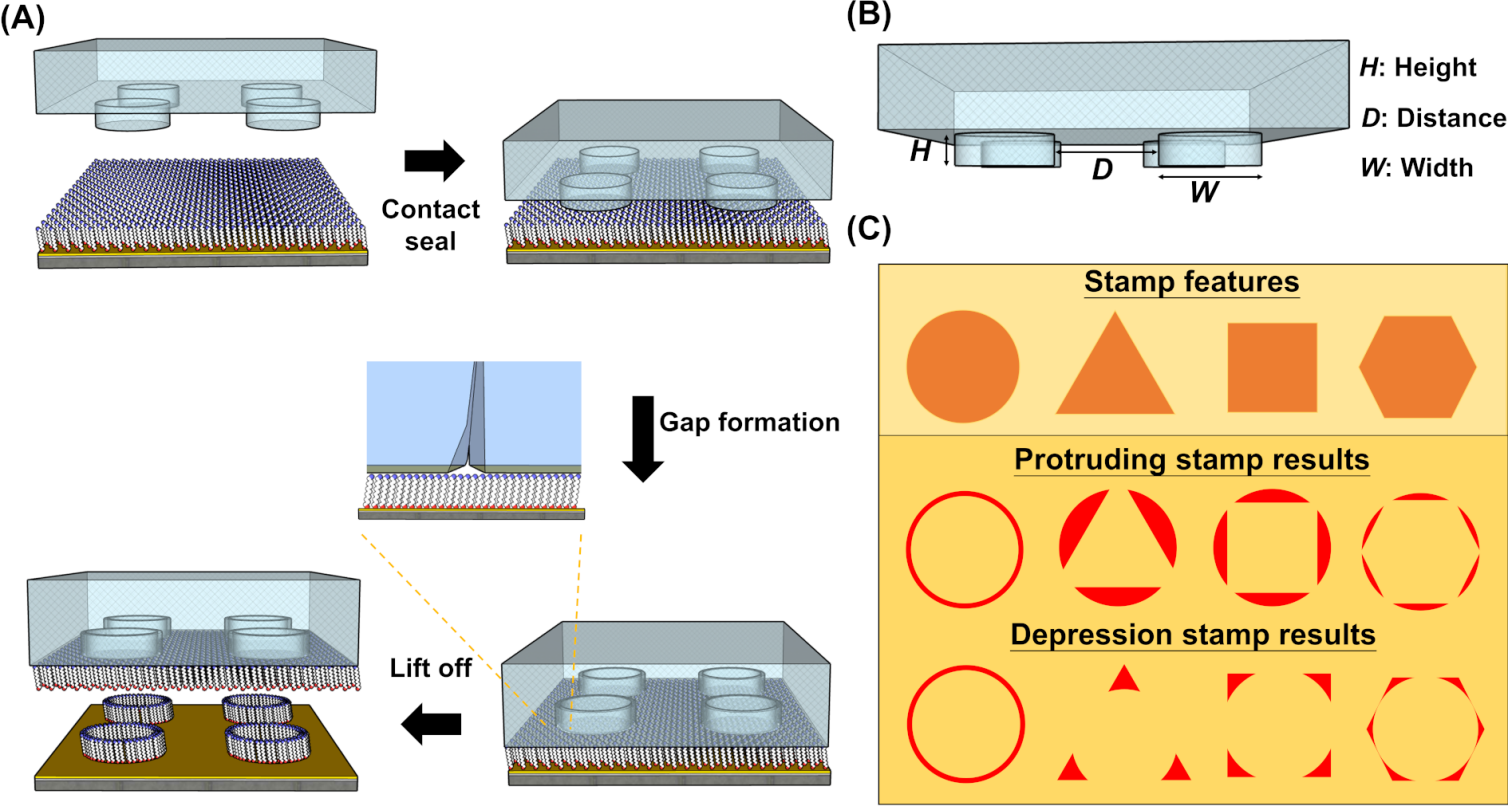
Illustration of the gap-directed chemical lift-off lithography process. (A) The selective removal of alkanethiols from an Au surface using a collapsed feature leaves extreme high resolution of molecular patterns on a substrate resembling the interfacial gaps. (B) Important structural parameters in a PDMS mold affecting the gap-directed CLL process. (C) Expected surface patterns creating by protruding and depression stamps in the gap-directed CLL. The yellow parts are contacted areas between the PDMS stamp and the SAM-covered Au, while the red regions indicate the gaps in between the stamp’s original feature and deformed parts. These red patterns are therefore the anticipated surface geometries after the gap-directed CLL operation.

Taking advantage of the diffusion-free CLL operation, here we demonstrate a straightforward method to generate unique chemical patterns by spontaneous structural gaps creation and further push CLL resolution to a new limit (Scheme 1A). The main concept of this approach lies on the deliberately-designed soft material stamp structure geometry and stereo dimension. To induce the spontaneous generation of controllable structure gaps at the stamp–substrate interface, the feature height (*H*), spacing distance (*D*), and width (*W*) on a soft material stamp are important parameters to modulate (Scheme 1B). *H* is regulated by the photoresist mold in which the PDMS resin is cured in, which in turn can be controlled by spin coating speed and photoresist concentration. On the other hand, *D* and *W* are both determined by the photomask used. It is important to note that the tiny gaps generated through soft material structure collapsing onto a flat substrate are located in between the original contact area and a later collapsing region. This newly formed space dimension should be much smaller than the feature size rendered on the original stamp, and this phenomenon can be exploited to achieve nanoscale patterns using microscale features on a stamp. Importantly, the lateral diffusion-free CLL process should maintain tiny gap features without hassles of random molecular movements and high resolution patterns are expected after this operation. We anticipate two types of molecular patterns to be produced via this approach. For a protruding stamp feature (hereafter referred to as a pillar), gaps between the pillar itself and a circular region slightly larger than the pillar are expected to form. On the other hand, gaps should show up between a depressing stamp feature (hereafter referred to as a hole) and the middle circular region slightly smaller than the hole structure (Scheme 1C). In a CLL operation, alkanethiols are removed wherever contact occurs between the SAM and the stamp structure, depicted by the color yellow in Scheme 1C. The newly formed gaps, depicted with the color red, are regions that the elastic stamp feature does not come into contact with molecules on the surface, thereby are still covered with alkanethiol molecules after stamp removal. Combining the merits of delicate molecular lifting off control in CLL and the unique surface patterns with sharp edges originating from mathematical geometry designs, the results of this study are expected to have deep implications in the facile fabrication of plasmonic structures, waveguides, and diverse nanostructures.

## Experimental

### Materials and chemicals

11-Mercaptoundecanol (MCU), triethylene glycol undecanethiol (TEG), and biotinylated alkane PEG thiol (BAT) were purchased from Sensopath Technologies Inc. (Bozeman, MT, USA). Streptavidin was purchased from Novus Biologicals (Centennial, CO, USA). FITC-labelled antistreptavidin antibody was purchased from Jackson ImmunoResearch Inc. (West Grove, PA, USA). Bovine serum albumin (BSA) and 10× phosphate buffered saline (PBS, contains 1.37 M NaCl, 0.027 M KCl, 0.10 M Na_2_HPO_4_, and 0.018 M KH_2_PO_4_) were purchased from Bioman Scientific Co., Ltd. (Taipei, Taiwan). Ethanol (99.5 wt %), acetone (99.5 wt %), and isopropanol (99.9 wt %) were purchased from Echo Chemical (Taipei, Taiwan). Hexamethyldisilazane (HMDS) was purchased from Sigma-Aldrich (St Louis, MO, USA). Iron nitrate and thiourea were purchased from Showa Chemical Industry Co., Ltd. (Tokyo, Japan). Positive photoresist AZ6112 was purchased from AZ Electronic Materials Taiwan Co., Ltd. (Taipei, Taiwan). T238 developer was purchased from Control Chemitech Inc. (Taoyuan, Taiwan). Silicon wafers were obtained from Mustec Corp. (Hsinchu, Taiwan). SYLGARD 184 silicone elastomer base and curing agent were purchased from Dow Corning Corp. (Midland, MI, USA). Deionized water (>18 MΩ·cm) was obtained from the ELGA PURELAB classic system (Taipei, Taiwan).

### CLL operation processes and bioactive substrates preparation

In a manner similar to a previous study [26], silicon substrates with 100 nm thick Au and 5 nm Cr adhesive layers were prepared by thermal evaporation. To prepare a SAM-covered Au substrate, the Au surface was immersed in a 2 μM ethanolic TEG thiol solution or 1 mM ethanolic MCU solution for overnight. After SAM formation, the substrates were washed with ethanol to remove excess thiol molecules and blown dry with nitrogen gas. To make PDMS stamps for CLL operation, a 10:1 mass ratio of SYLGARD 184 silicone elastomer base and curing agent were thoroughly mixed with a homogenizer, degassed in vacuum, cast onto the silicon master mold created through photolithography, and cured in an oven at 80 °C for 1 h. The PDMS stamps were then separated from the master mold, sequentially rinsed with acetone and isopropanol, and blown dry with nitrogen gas. To conduct CLL processes, a PDMS stamp was activated by 30 s of oxygen plasma exposure at a power of 18 W with 0.5 mbar oxygen flow. The stamp was then conformally sealed onto the SAM-modified Au substrate for 2 h under ambient environment without additional compression force. The collapse of stamps occurs spontaneously and is dependent on the properties of the PDMS stamp. Thereafter, the contact-sealed stamp was removed from the Au substrate, and the Au surface was immersed in a 1 mM ethanolic BAT solution to backfill ligand molecules into the post-chemical lift-off regions. After 1 h of incubation, the substrate was washed with ethanol, treated with BSA (10 mg/mL in PBS buffer) for 5 min, transferred into a streptavidin solution (50 μg/mL in PBS buffer) for 20 min, followed by FITC-labeled anti-streptavidin antibody solution (10 μg/mL in PBS buffer) incubation for 20 min to complete the biorecognition process. Finally, the substrate was gently rinsed by deionized water, immersed in PBS buffer solution, and stored at 4 °C in the dark before further characterization. Fluorescence and bright filed microscopic images were captured with an epifluorescence microscope (Axio Imager. M2, Carl Zeiss microscopy, Jena, Germany) equipped with an X-Cite 120 LED (Lumen Dynamics Group, Inc., Mississauga, Canada) lamp. Excitation wavelength was set to 480 nm while emission of 535 nm was collected. Fluorescence images were analyzed with the rectangle function in ZEN 2012 Service Pack 2 software (Carl Zeiss Microscopy, Jena, Germany). XPS spectra after each surface modification step were collected with ULVAC-PHI X-ray photoelectron spectrometer (PHI QuanteraII, Kanagawa, Japan).

### Selective wet chemical etching processes and metal structure characterization

To transfer chemical patterns created by CLL to the underneath metal layer, a wet chemical etching process was adopted. After lifting the PDMS stamp from a SAM-modified Au substrate, the Au surface was immersed in an aqueous mixture containing 40 mM iron nitrate and 60 mM thiourea to etch the exposed underlying Au film. After 30 min of etching, the substrate was rinsed with deionized water and blown dry with nitrogen gas. The transferred metal structures were then characterized by optical microscopy, scanning election microscopy (SEM, JEOL JSM-7600F, Tokyo, Japan) and atomic force microscopy (Dimension Fastscan, Bruker Nano Surfaces, Hsinchu, Taiwan).

## Results and Discussion

**Figure 1:**
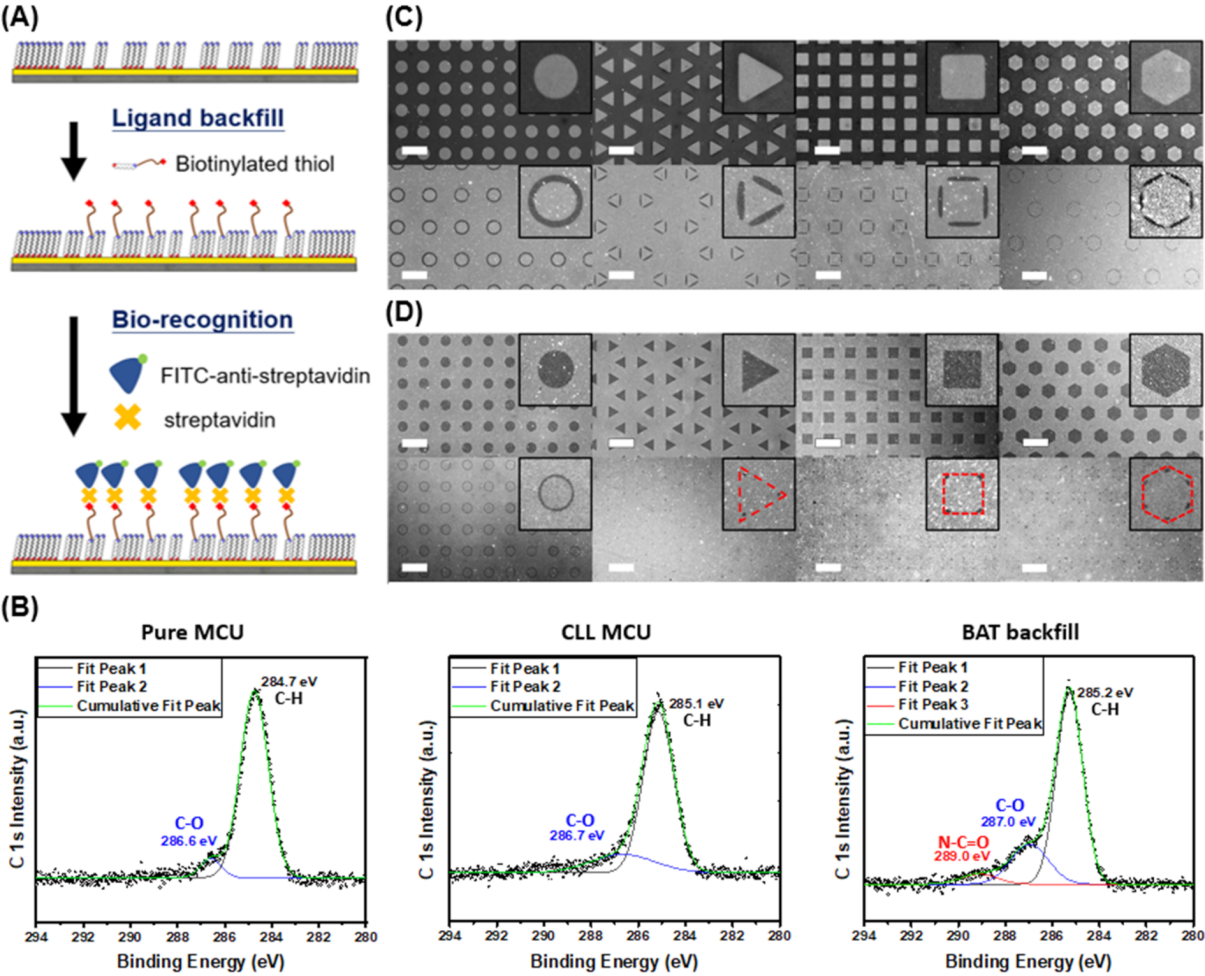
Visualization of surface patterns created by gap-directed CLL through biorecognition. (A) Schematic illustration of the biorecognition process including post lift-off region biotinylated thiol backfilling, streptavidin binding, and antibody recognition. (B) XPS characterization of the surface for (left) pure MCU SAM, (center) MCU SAM after CLL treatment, and (right) after BAT backfill. Fluorescence images of biotin–streptavidin biorecognition on different CLL-produced geometric patterns made with (C) protruding PDMS stamps: non-collapsed (top), collapsed (bottom) and (D) depression PDMS stamps: non-collapsed (upper), collapsed (below). The scale bars are 100 μm.

The results of selective SAM removal are visualized by backfilling biotinylated alkanethiol (BAT) molecules into the post lift-off regions followed by conjugating streptavidin and FITC-labeled anti-streptavidin antibodies to the biotin moieties. In these tests, 11-mercaptoundecanol (MCU) molecules on an Au surface are selectively removed by the oxygen plasma activated PDMS stamp at stamp-substrate contact regions. BAT molecules are thereafter backfilled into the exposed surface vacancies, and the biotin moieties are conjugated with streptavidin followed by FITC-labeled anti-streptavidin antibody recognition (Figure 1A). This process results in obvious contrast in fluorescence images, allowing the visualization of biorecognition processes at post lift-off regions. XPS spectroscopy is utilized to monitor the surface chemical composition after each intermediate step in the CLL process (Figure 1B). As expected, prominent peaks indicating the presence of C–H and C–O bonds can be observed for substrates covered with MCU SAM, and a spectrum with similar peaks is obtained after partial SAM removal with an activated PDMS stamp. The presence of BAT molecules after backfilling is confirmed via the observation of a new C–N signal in the spectrum. For CLL performed with pillar-featured PDMS stamps, the roof collapse phenomenon is determined by the distance between pillars (*D*) when the other two parameters (*W* and *H*) are fixed. As demonstrated in Figure 1C, the top row images reveal biorecognition results on a post lift-off surface when no PDMS stamp collapse happens in the CLL process. Comparably, collapsed stamp structures result in completely different patterns shown in the bottom row when the inter-pillar distance is increased. When stamps with hole structures are used in the CLL process, an inversed biorecognition pattern is expect as demonstrated in Figure 1D. Similarly, collapse-free and collapsed stamp structures result in different pattern geometries on the surface as shown in top and bottom row images, respectively. Since the stamp hole structure inter distance does not affect the collapse phenomenon, less stiff PDMS stamps are created by reducing the overall stamp thickness to induce roof collapse in the CLL operation. It is important to note that both the pillar and hole structures produced post lift-off surface biorecognition geometries are in line with the predictions shown in Scheme 1C: the gap of pillars and their circumscribed circles are retained for protruding stamps, while the depressed stamps resulted feature resembles the gap between the holes’ outer parameter and their inscribed circles. It is also worth noting that about only one tenth the size of original features at the shapes’ vertices are preserved for triangular, square, and hexagonal hole stamps due to the soft nature of the PDMS stamps.

**Figure 2:**
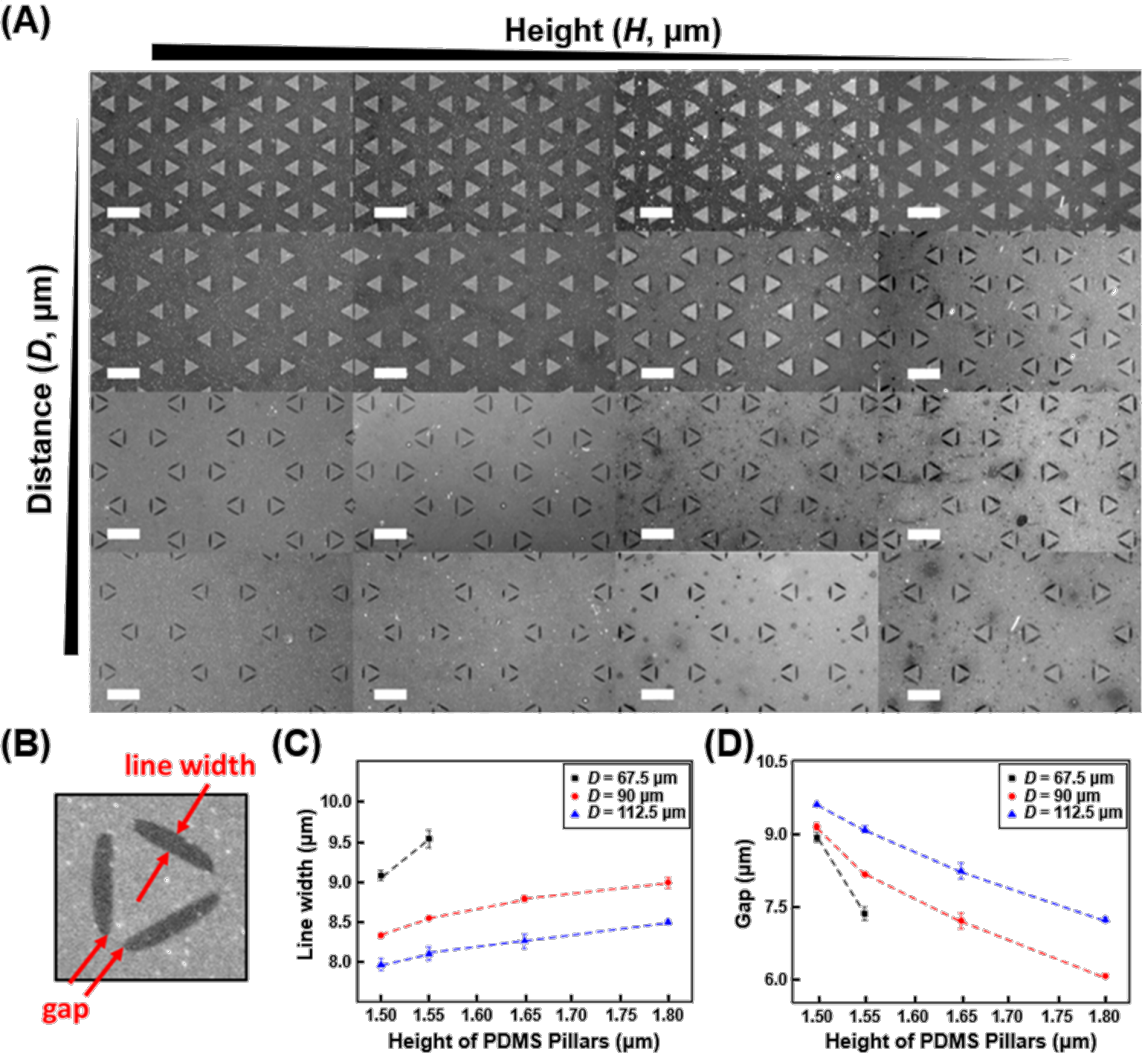
Controls over rubber stamp structural parameters for gap-directed CLL and the resulted fluorescent biorecognition surface patterns. (A) Fluorescence images of biotin–streptavidin recognition on post lift-off regions using stamps rendering identical sized triangular pillars with different pillar heights and inter pillar distances. The scale bars are 100 μm. (B) Definition of surface generated patterns (line width and gap) in a triangular feature. (C) and (D): Effects of the PDMS pillar structure height (*H*) and inter pillar distance (*D*) on produced feature line width and gap. (*N* = *6*) (Note: Only two data points are reported for *D* = 67.5 μm in (C) and (D) because the pillars do not collapse, thus no line width and gap values could be reported.)

The stamp roof collapsing effect is controllable and would lead to different contact regions in the CLL process, which should result in various post lift-off biorecognition patterns on a surface. This alteration tendency can be observed when the pillar height (*H*) and the inter pillar distance (*D*) parameters are tuned with a fix of geometry width (*W*). Stamps with triangular pillars rendering the following parameters are evaluated for this demonstration: *W* = 52.0 μm; *H* = 1.80 μm, 1.65 μm, 1.55 μm, and 1.50 μm; *D* = 45.0 μm, 67.5 μm, 90.0 μm, and 112.5 μm. (*W* refers to the triangle side length, *D* is the side-to-side distance between two adjacent triangles, and *H* stands for the triangular pillar height.) As shown in Figure 2A, similar biorecognition process produced fluorescence images are collected when stamps with the aforementioned variables are applied in the CLL operation. It should be noted that the surface patterns observed here rely on whether tiny gap spaces are created between the original stamp contact area and a latter collapsed region. In these fluorescence images, the lower contrast areas represent places where no biorecognition happens after the CLL operation. It is clear to see that a reduced pillar height induces a more obvious collapsing phenomenon when the inter pillar distance is fixed. Comparably, stamp structure collapsing is enhanced when the pillar side-to-side distance is increased under a maintained feature height. Manipulation of these two stamp structural parameters allows the control of generated surface pattern geometries (Figure 2B–D). As revealed in Figure 2C, the feature line width (defined as the crest shape width at its thickest part) reduces with a lower PDMS pillar height due to the enhanced collapsing effect. Meanwhile, the shrinkage of a crest shape accompanies a larger necklace geometry gap (defined as the distance between two crest vertexes) formation. Since the change of line width and necklace gap depends on the magnitude of collapsing phenomenon, similar line width reduction and gap enlarging effects are also observed when the inter pillar distance is raised. Previous studies have shown that the roof-collapse phenomenon is affected by four parameters, *D*, *H*, *W*, and the elastomer’s Young’s modulus [40,46]. In current experimental setups, *D* and *H* are used to tune the formation of self-collapse-induced structural gaps, but it is reasonable to assume that both *W* and the elastomer stamp’s physical properties would have impacted the result as well. Increasing *W* or utilizing stamps with greater Young’s modulus (stiffer) while fixing other parameters would decrease the tendency for roof to collapse, thus increasing line width and decreasing gap size.

**Figure 3:**
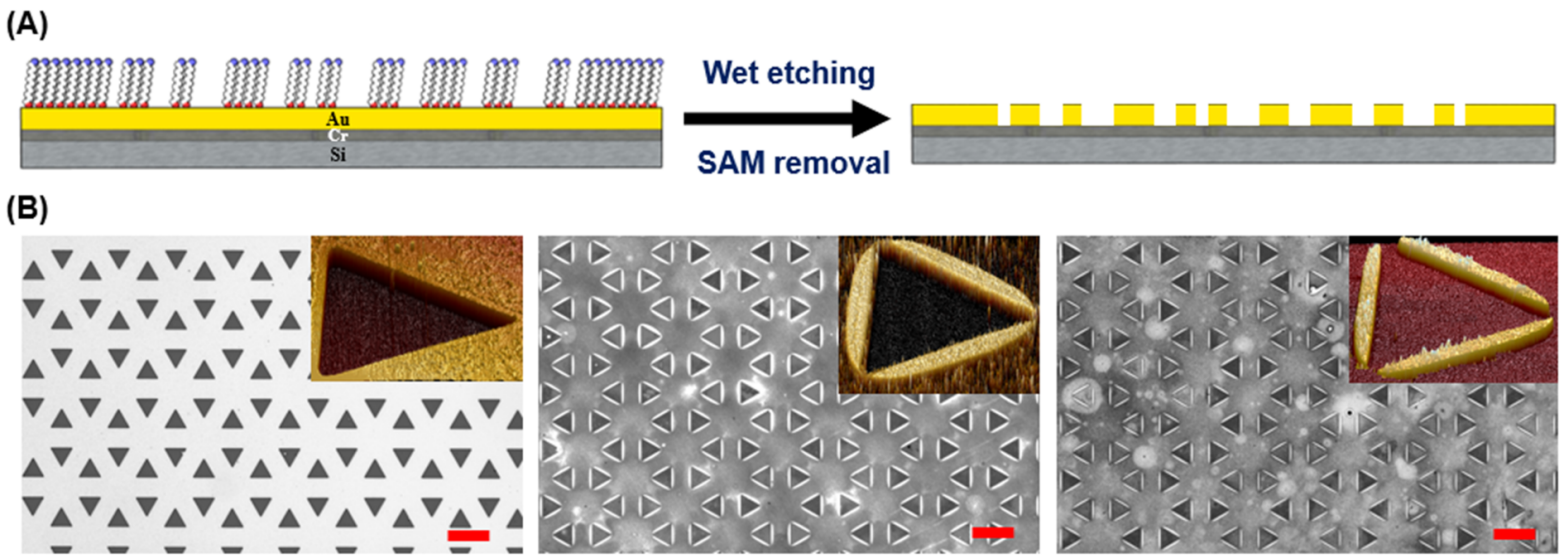
The transfer of CLL created molecular pattern to the underneath Au layer through wet chemical etching. (A) Schematic illustration of the post lift-off wet etching process. (B) Bright field optical microscope and atomic force microscope topography images (inset) of Au structures fabricated by gap-directed CLL using collapse-free (left) and self-collapsing (middle and right) stamps rendering triangular pillars followed by wet etching. Uneven regions are due to the reduced wet etching time used in order to preserve the tiny gold structures. Stamp heights are 2.25 μm (left), 2.00 μm (middle), and 1.50 μm (right). Scale bars are 100 μm.

In addition to acting as the matrix for biorecognition arrays, the molecular pattern created on a post lift-off surface can serve as the molecular resist for the underneath material structure transfer. This concept is demonstrated by immersing a post-lift off surface in an aqueous mixture containing iron nitrate and thiourea for wet chemical etching (Figure 3A). The absence of SAM molecule protection at the stamp contact area after CLL treatments allows the replication of molecular features to the Au layer. As shown in Figure 3B, various Au structures are obtained when stamps rendering different heights of triangular pillars are used in the lift-off process. With collapse-free pillar structures (*H* = 2.25 μm), inversed triangular metal hole features are created on the Au layer. Along with pillar height reduction (*H* = 2.00 and 1.50 μm), Au structures similar to predictions in Scheme 1C are obtained. It should be noted that both the original contact and collapse-induced contacting areas remove surface alkanethiol molecules and expose the underneath Au layer to chemical etchants. Unique connected or separated necklace-like metal geometries are therefore obtained depending on the magnitude of stamp collapsing onto the supporting substrate.

**Figure 4:**
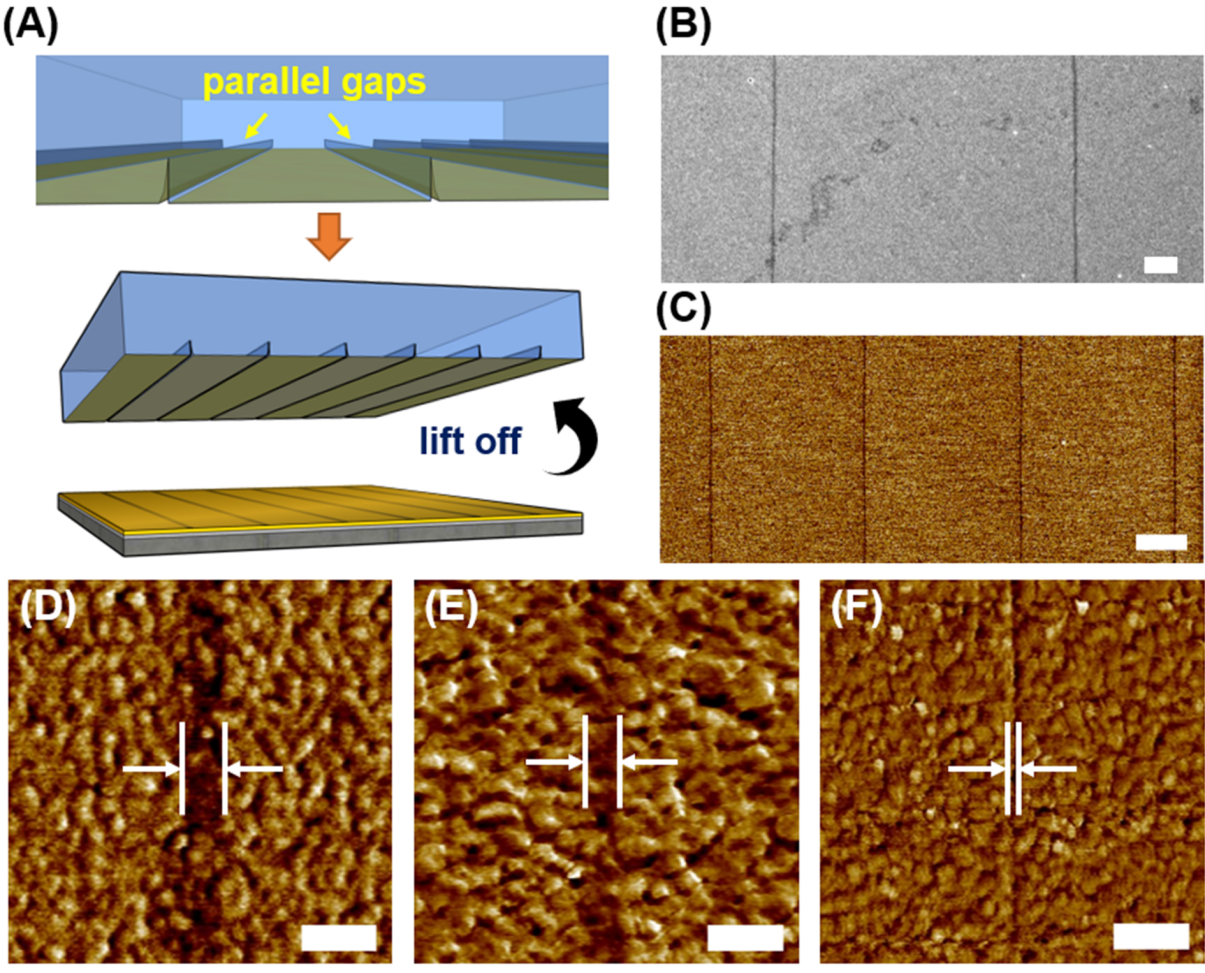
Gap-directed CLL operated with microscale parallel line shape features (*W* = 6 μm, *D* = 6 μm) and the resulted nanoscale lines. (A) Schematic illustration of line gaps created by a stamp with parallel line features when the roof collapses onto a surface. Alkanethiol molecules are therefore selectively removed except to the gap areas in a CLL process. The resulted features are characterized by SEM for feature line width of (B) 150 nm and by AFM for feature line widths (C) 80 nm (D) 50 nm (E) 35 nm, and (F) 5 nm. Scale bar is 2 μm (B) and (C), and 100 nm (D–F).

Based on this gap-directed CLL operation, surface features that are at least an order of magnitude smaller than the original stamp structures can be obtained. This is attributed to the combination of the lateral diffusion free property of CLL and the minute structural gap formation in the feature transfer process. We envision the employment of this concept on advancing current CLL process resolution through straightforward experimental designs, i.e., interfacial gap creation. To demonstrate the gap manipulation on CLL process capability improvements, a lowered stamp feature height is employed while other structural parameters are fixed. PDMS stamps with micrometer-scale width parallel line patterns (*W* = 6 μm, *D* = 6 μm) featuring different heights are produced to initiate different levels of spontaneous collapsing in the CLL operation (Figure 4A). Through this design, the collapsed stamp roof creates parallel line shape gaps between two adjacent stamp line features. These gaps therefore result in parallel linear SAM strips ranging from 150 nm to sub-10 nm wide after the CLL operation, and a new resolution limit of 5 nm in the CLL process is achieved when the used stamp height is 700 nm (Figure 4B–F). For 150 nm features SEM (JEOL JSM-7600F Schottky Field Emission Scanning Electron Microscope, Tokyo, Japan) is used, but for sub-100 nm features AFM is employed to avoid the heavy influence of high-energy electron beams on molecular patterns in imaging. The straightness of the obtained lines is also assessed by calculating the linear regression of pattern edges. Coordinates of 10 evenly spaced points on edges of line features are extracted and a regression line is obtained. The R^2^ values are calculated to be at least 0.9858 for all line borders shown in the AFM images of Figure 4, indicating the straight pattern edges and faithful transfers of features from the PDMS stamp are achieved. For reference, a comprehensive comparison between past works on SAM patterning techniques and this work is also included (Table 1).

**Table 1:** Comparison of recent techniques on SAM patterning.

Patterning strategy	Resolution	Reference

*contact modification*		
substitution of a bromo-terminated monolayer with NaN_3_	3 μm	[47]
photochemical reaction of tetrazoles to form carboxylates	unspecified	[48]

*non-contact modification*		
octadecylsiloxanes modified with electron beams	5 nm	[49]
UV conversion of nitrobiphenylthiol nitro groups to amino groups and cross-linking of aromatic groups	20 nm	[50]
UV degradation of oligo(ethylene glycol)-functionalized trichlorosilanes	30 nm	[51]
exposure of alkanethiolates to UV laser followed by thiol immersion	100 nm	[52]

*stamp additive*		
contact/non-contact printing of hydrophilic ink and hydrophobic ink, respectively, with PDMS stamp	20 μm	[53]
transfer of alkanethiolates from PDMS stamp to surface	200 nm	[54]

*stamp subtractive*		
removal of alkanethiols at PDMS stamp contact regions to the surface	40 nm	[17]
removal of alkanethiols at PDMS stamp contact regions to the surface	sub-30 nm	[27]

*tip additive*		
transfer of 1-octadecanethiol from AFM tip to surface through water meniscus	30 nm	[55]
transfer of 16-mercaptohexadecanoic acid from AFM tip to surface through water meniscus	5 nm	[56]

*tip subtractive*		
removal of bovine serum albumin with AFM tip	15 nm	[57]
removal of hydroxy-terminated alkanethiol with sharp PDMS stamp	sub-20 nm	[58]

## Conclusion

In summary, we demonstrate a straightforward method to reliably produce surface features that are orders of magnitude smaller than the original stamp structures. This approach utilizes structural gaps generated between the original rubber stamp contact region and a later collapsed area to initiate a gap-directed CLL process for arbitrary surface patterning. Importantly, nanometer-scale surface features can be obtained from micrometer-scale stamp features, thus greatly lowering the technical barrier in lithographic patterning. Diverse patterns, including lines, circles, triangles, squares, hexagons, and arrayed dots sized down to 5 nm can be obtained by implementing PDMS stamps with corresponding features and varying dimensions. This achievement exceeds the conventional CLL minimum feature width of 15 nm and sub-30 nm obtained using double lift-off and stamp self-collapsing techniques, respectively. In addition to molecular patterning for biorecognition, the obtained post lift-off patterns can be accurately transferred to the underlying Au substrate for metallic structure creation. Relying on the integration of delicate stamp collapsing controls and the lateral diffusion-free CLL operation, this strategy produces unique necklace-like structures rendering high resolution and sharp edges. They can be transferred to other substrates for useful applications such as plasmonic signal enhancement, waveguides, and nanopores with specialized designs. This method is a large improvement over conventional soft lithographic techniques as the employment of minute gaps in collapsing stamps allow even finer patterns to be fabricated with inexpensive photomasks. With this technology, we foresee that the straightforward generation of versatile nanoscale patterns can further push the boundaries of CLL, and expand its applications in solving conventional biosensing, nanoelectronics, and semiconductor problems.
